# Tau processing and tau-mediated inflammation differ in human *APOEε2* and *APOEε4* astrocytes

**DOI:** 10.1016/j.isci.2024.111163

**Published:** 2024-10-11

**Authors:** Tobias Mothes, Evangelos Konstantinidis, Khalid Eltom, Abdulkhalek Dakhel, Jinar Rostami, Anna Erlandsson

**Affiliations:** 1 Uppsala University, Department of Public Health and Caring Sciences, Molecular Geriatrics, Uppsala, Sweden

**Keywords:** disease, genes, neuroscience

## Abstract

Alzheimer’s disease (AD) and progressive supra-nuclear palsy (PSP) are both proteinopathies, characterized by the accumulation of tau aggregates. *APOEε4* is the greatest genetic risk factor for developing AD, while *APOEε2* is a significant risk factor for developing PSP. In the brain, astrocytes are the predominant producer of ApoE, but they are also important for inflammation and overall brain homeostasis. Although, tau inclusions appear frequently in astrocytes in both AD and PSP brains, their connection to ApoE remains unclear. Here, we show that hiPSC-derived *APOE 2/2* astrocytes accumulate, process, and spread pathogenic tau aggregates more efficiently than isogenic *APOE 4/4* astrocytes. Moreover, the *APOE 2/2* astrocytes display a more robust inflammatory response, which could be of relevance for the disease course. Taken together, our data highlight a central role of ApoE in astrocyte-mediated tau pathology.

## Highlights

• *APOEε2* promotes increased tau accumulation in human astrocytes, compared to *APOEε4*
• *APOEε2* and *APOEε4* astrocytes differ regarding tau modification and spreading
• *APOEε2* astrocytes show a more robust inflammatory response than *APOEε4* astrocytes
• There is no difference in neurotoxicity between *APOEε2* and *APOEε4* astrocytes

## Graphical abstract

**Figure:**
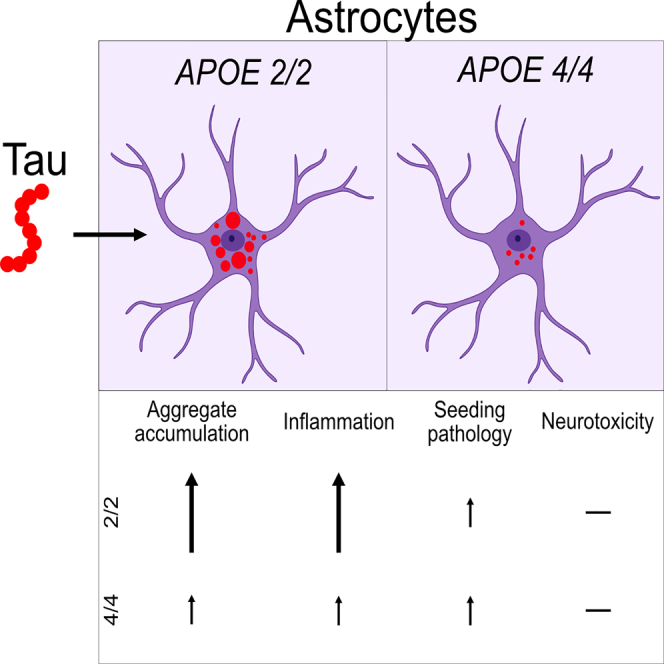

## Introduction

Alzheimer’s disease (AD) and other tauopathies are defined by the presence of pathological tau deposits in neurons, which are believed to spread from one cell to another in a prion-like manner. Tau inclusions are also frequently found in astrocytes, but their relevance for disease progression appears complex and varies between diseases.^1^^,^^2^^,^^3^ Being the most abundant glial cell, astrocytes play an important role in maintaining brain homeostasis. Their functions include metabolic support of neurons, modification of synapse signaling, recycling of neurotransmitters, blood-brain barrier regulation, and glymphatic clearance.^4^ Moreover, astrocytes play a critical role in lipid metabolism and are the primary apolipoprotein E (ApoE) producing cells in the brain.^5^

In humans, there are three ApoE isoforms; ApoEε2, ApoEε3 and ApoEε4. Carrying the *APOE*ε4 allele is the strongest genetic risk factor for developing sporadic AD. Individuals homozygous for *APOEε4* have a 8– to 16-fold increased risk of developing sporadic AD, compared to *APOEε3* carriers,^6^^,^^7^ while individuals homozygous for *APOEε2* have a reduced risk. On the other hand, *APOEε2*-carriers have an elevated risk of developing other diseases, including progressive supranuclear palsy (PSP), argyrophilic grain disease (AGD), cerebral amyloid angiopathy (CAA), and certain types of hyperlipidemia.^8^^,^^9^^,^^10^^,^^11^^,^^12^^,^^13^ Notably, the primary tauopathy PSP is defined by the presence of tau-immunoreactive astrocytes known as tufted astrocytes (TA).^14^ However, it remains unclear how the TA and other types of tau-containing astrocytes influence the disease progression. Furthermore, the *APOE* loss-of-function R154S mutation (Christchurch) highlights the link between tau pathology and *APOE* genotype, as the patient displayed excessive levels of amyloid pathology, but very low levels of tau and as a result only developed mild dementia late in life.^15^ Recently, several other *APOE* loss-of-function variants have been reported which are associated with resistance to AD.^16^ However, the underlying cellular mechanisms by which ApoE affect tau pathology is still not known.

Growing evidence indicates that the insufficient degradation of protein aggregates and chronic inflammation are driving forces of AD pathology.^17^^,^^18^^,^^19^ Consequently, the role of glial cells in disease progression has received much more attention. Our previous data show that astrocytes take up large amounts of aggregated tau, but are unable to successfully degrade the material. Instead, the astrocytes modify the internalized tau aggregates and act as an intermediator in the spreading of pathological tau species.^20^ The aim of this study was to investigate the impact of the PSP risk allele (*APOEε2*) and the AD risk allele (*APOEε4*) on astrocytic tau accumulation, tau spreading, tau-mediated inflammation and neurodegeneration, using isogenic human iPSC derived astrocytes.

## Results

### Both *APOE 2/2* and *APOE 4/4* astrocytes engulf aggregated tau

**Figure 1:**
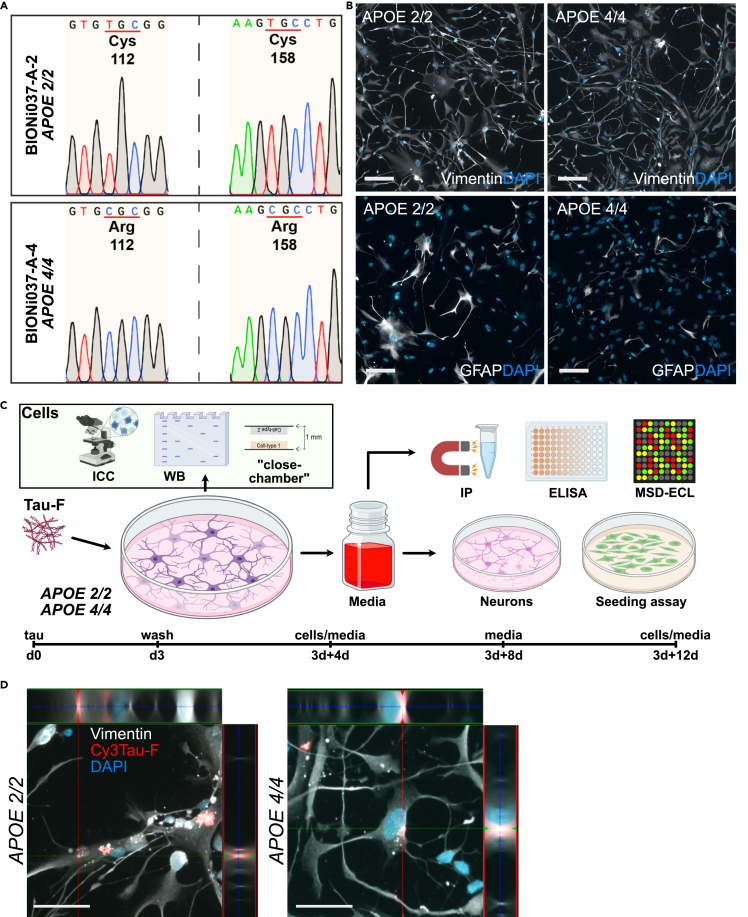
Characterization of *APOE 2/2* and *APOE 4/4* astrocytes and the experimental setup (A) DNA analysis confirming successful gene editing of BIONi308-A-2 (*APOE 2/2)* and BIONi037-A-(*APOE 4/4*) cells. (B) Example images of control *APOE 2/2* and *APOE 4/4* astrocytes, stained for vimentin and GFAP. Scale bars = 100 μm. (C) Schematic outline of the experimental setup. Isogenic human iPSC derived astrocytes (*APOE 2/2* and *APOE 4/4*) were exposed to synthetic tau fibrils (Tau-F) for 3 days. Then, the astrocytes were washed and further incubated in a tau-free medium for 4–12 days. Cells were fixed or lysed at 3days+4days and 3days + 12days and medium was collected at 4, 8, and 12 days post-wash. The cells were analyzed using immunocytochemistry, Western blot and cultured in a close-culture setup together with *APOE 3/3* astrocytes. The medium was directly analyzed using IP, ELISA and a cytokine array (MSD-ECL); or added to iPSC derived neuronal cultures or HEK293T RD tau FRET Biosensor c (seeding assay). (D) z stack of Cy3Tau-F inclusions inside *APOE 2/2* and *APOE 4/4* astrocyte at 3days+4days, vimentin (white), tau (red) and DAPI (blue). Scale bar = 50 μm.

To elucidate the relevance of astrocytic *APOE* genotype on tau pathology we utilized isogenic CRISPR/Cas9 modified iPSC lines to generate *APOE 2/2* and *APOE 4/4* astrocytes, following a previously described protocol (Figure 1A).^21^ Successful astrocytic differentiation was confirmed with western blot analysis of GFAP and S100β (Figure S1). Importantly, the *APOE 2/2* and *APOE 4/4* astrocytes displayed similar morphology and expression levels of vimentin and GFAP, indicating that the APOE genotype did not adversely affect the differentiation process (Figure 1B). To study the effect of tau pathology over time, the astrocytes were exposed to synthetic tau fibrils (Tau-F) for 3 days, after which the cultures were thoroughly washed and cultured for up to 12 days in tau-free medium. Both the medium and the cells were analyzed at 3days+0days, 3days+4days, 3days+8days, and 3days + 12days, using a battery of different methods (Figure 1C). The astrocytes were analyzed using immunostainings and Western blot analysis, as well as a custom “close-culture” chamber together with a separate line of *APOEε3* astrocytes (iPSC line, Cntrl9 II). The astrocyte medium was analyzed using ELISA, cytokine array, tau seeding assay, and toxicity studies in human neuronal cultures. Confocal imaging demonstrated that both *APOE 2/2* and *APOE 4/4* astrocytes effectively ingested Cy3-labelled Tau-fibrils (Figure 1D). Deposits of internalized tau appeared predominantly to be stored in aggrosomes, identified by their dense cage-like vimentin network (Figures S2A and S2B). However, some of the smaller tau aggregates were situated inside LAMP-1 positive lysosomes, indicating that the astrocytes made efforts to degrade these inclusions (Figure S2C). However, we did not detect any apparat difference in the number of Cy3-tau positive puncta, situated within lysosomes between the two genotypes.

### Astrocytic *APOE*-genotype drastically affects the cellular processing of ingested tau fibrils

**Figure 2:**
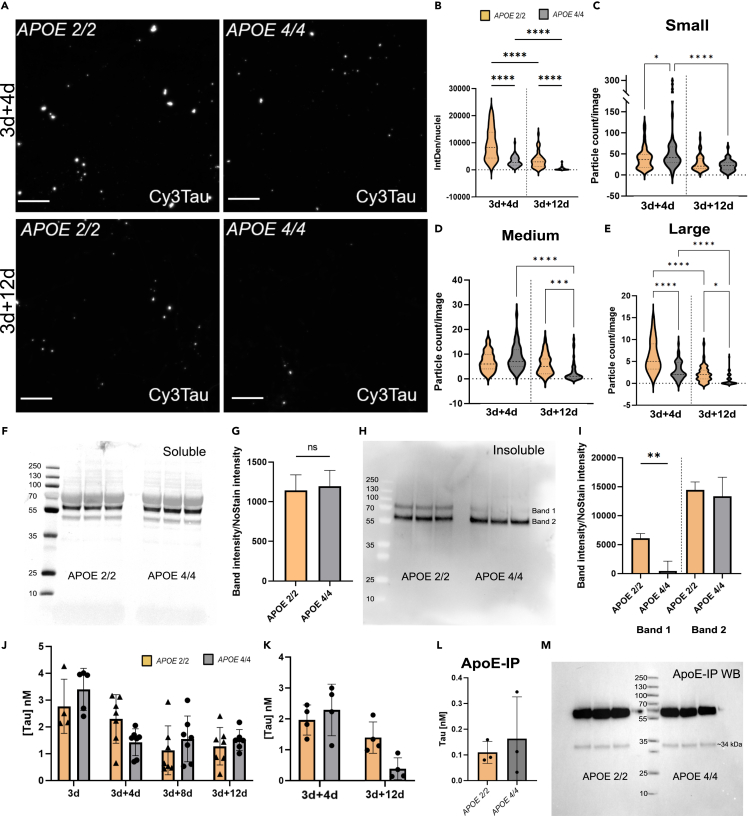
APOE genotype drastically affects processing of internalized tau protein in iPSC derived astrocytes (A) Representative images of Cy3 labeled Tau-F internalized by APOE 2/2 and APOE 4/4 astrocytes at 3 + 4 and 3 + 12 days (The same images, including membrane dye and DAPI are shown in Figure S3). Scale bars = 100 μm. (B) Quantification of intracellular Cy3Tau-F IntDen, normalized to number of nuclei per field. Analyzed using one-way ANOVA. (C–E) Number of small, medium, and large Cy3Tau-F inclusions inside APOE 2/2 and APOE 4/4 astrocytes at 3days+4days and 3days + 12days. Small aggregates = 0.53–21.1 μm^2^, medium aggregates = 21.2–105.6 μm^2^ and large aggregates = 105.7–1056 μm^2^. (F) Total tau (Tau-12, Tau-5, BT2, and T46) Western blot analysis of the soluble fraction of lysed APOE 2/2 and APOE 4/4 astrocytes at 3 + 12days. (G) Quantification of the WB in f. Band intensity was normalized to the band intensity of the NoStain blot (Figure S5). Analyzed using a standard t-test. (H) Total tau (Tau-12, Tau-5, BT2, and T46) western blot of the insoluble fraction from APOE 2/2 and APOE 4/4 astrocytes lysates after 3 + 12days exposure. Band 1–75 kDa; Band 2–60 kDa. (I) Quantification of WB in h. Band intensity was normalized to the band intensity of the NoStain blot (Figure S5). Analyzed using a standard t-test. (J) Sandwich ELISA analysis of ACM from APOE 2/2 and APOE 4/4 astrocytes at the different time points (3days+4days, 3days+8days and 3days + 12days). (K) Sandwich ELISA analysis of lysates from APOE 2/2 and APOE 4/4 astrocytes. (L) Tau concentration measured by ELISA in the ApoE-IP fraction of ACM from Tau-F exposed APOE 2/2 and APOE 4/4 astrocytes. (M) WB of ApoE-IP fraction stained using the same ApoE antibody (NoStain blot in Figure S5). Data is presented as mean ± SD. P-values are presented as following; ∗*p* < 0.05, ∗∗*p* < 0.01, ∗∗∗*p* < 0.005, ∗∗∗∗*p* < 0.0001.

In previous publications, we have shown that both murine and human astrocytes store, rather than fully ingested protein aggregates of tau, α-synuclein, and amyloid-β.^20^^,^^22^^,^^23^^,^^24^^,^^25^^,^^26^^,^^27^^,^^28^ Hence, we sought to compare the intracellular tau deposits in *APOE 2/2* respective *APOE 4/4* astrocytes over time. Interestingly, there was a substantial difference in intracellular Cy3Tau-F between the two *APOE* genotypes (Figures 2A and S3). Quantification of intracellular Cy3Tau-F revealed a much greater signal in *APOE 2/2* astrocytes compared to *APOE 4/4* astrocytes, which was consistent over time (Figure 2B). Notably, the *APOE 4/4* astrocytes showed virtually no Cy3-positivity at the latest time point. Inclusions in *APOE 2/2* appeared to be both larger and more distinct (Figures 2C–2E). Quantification of the number of tau deposits/field confirmed that there was a significant decrease in both small (Figure 2C), medium sized (Figure 2D), and large inclusions (Figure 2E) in *APOE 4/4* astrocytes over time, while there was only a modest decrease in large inclusions in *APOE 2/2* astrocytes (Figures 2C–2E). This highlights a discrepancy in protein storage between the two genotypes. It is worth noting that *APOE 4/4* astrocytes proliferated to a greater extent than the *APOE 2/2* astrocytes (Figure S4), which could affect the tau deposits/cell (Figure 2B). However, the proliferation would not have an impact on the tau deposits/field (Figures 2C–2E). Considering the stark difference in tau accumulation, we next investigated whether the *APOE* genotype influenced the degradation of the ingested tau fibrils by analyzing the soluble and insoluble cell lysate fractions. WB of the soluble fraction indicated no difference in intracellular tau between *APOE 2/2* and *APOE 4/4* astrocytes at 3days + 12days (Figures 2F and 2G). However, analysis of the insoluble fraction revealed a clear difference between the two genotypes. While the *APOE 2/2* astrocytes showed two distinct bands for tau (Band 1–75 kDa and Band 2–60 kDa), *APOE 4/4* astrocytes showed only a single band (Band 2–60 kDa) (Figure 2H). Quantification of the band intensity verified that there was no difference in the amount of tau that appeared at 60 kDa, but a dramatic difference in the amount of tau that appeared at 75 kDa (Figure 2I). This result demonstrates that the *APOE* genotype affects the cellular processing of engulfed tau fibrils. To corroborate these findings we performed tau ELISA on lysates and the astrocyte-conditioned media (ACM). The relatively low levels of tau detected in the medium at day 3 (prior to the washing step) show that the astrocytes internalized the majority of protein present in the medium over the three days. Furthermore, the levels of tau in *APOE 2/2* and *APOE 4/4* ACM were similar at the different time points after the washing step, suggesting that there were no differences in the secretion of tau aggregates between the genotypes (Figure 2J). Analysis of the tau levels in cell lysates shows a similar result as the ICC data, with higher concentrations of intracellular tau in *APOE 2/2* astrocytes, compared to *APOE 4/4* astrocytes (Figure 2K). Others have reported that ApoE 2 but not ApoE 4 can bind and form complexes with tau *in vitro*. To verify if this was the case in our cell system, we performed immunoprecipitation (IP) of ACM, using an ApoE antibody. The IP samples were then analyzed with tau-ELISA to confirm the presence of ApoE-tau interactions. Interestingly, we detected low levels of tau in the ApoE-IP fraction from both *APOE 2/2* and *APOE 4/4* ACM, indicating that ApoE-tau complex formation does occur in a biological setting (Figure 2L). However, there was no difference between *APOE 2/2* and *APOE 4/4* astrocytes and the total ApoE levels were equivalent between the genotypes (Figure 2M).

### APOE genotype influences the astrocytic inflammatory profile

**Figure 3:**
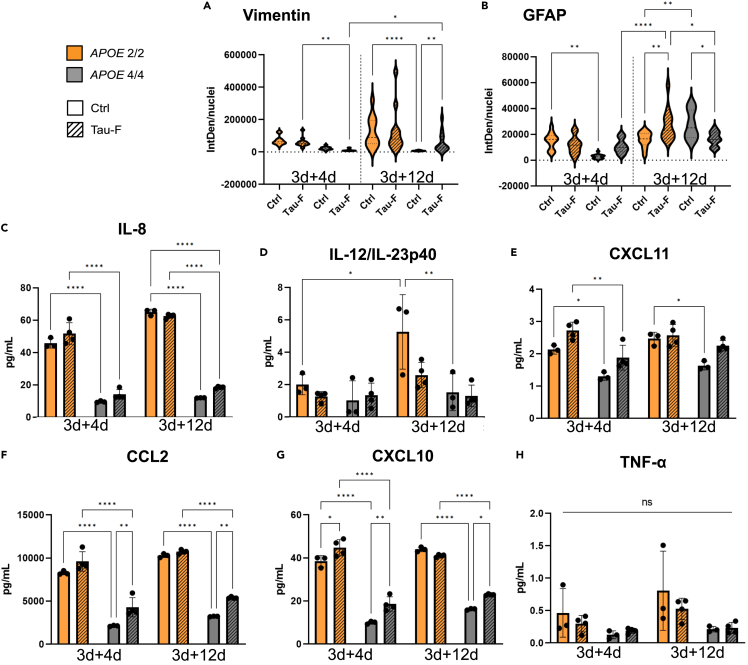
APOE genotype influences the inflammatory response of iPSC derived astrocytes (A) Quantification of vimentin expression in APOE 2/2 and APOE 4/4 astrocytes, normalized to the number of nuclei per field. (B) Quantification of GFAP expression in APOE 2/2 and APOE 4/4 astrocytes, normalized to the number of nuclei per field. (C–H) Cytokine levels in ACM from control/Tau-F exposed APOE 2/2 and APOE 4/4 astrocytes as measured by U-Plex MesoScale. (C) IL-8. (D) IL-12/IL-23p40. (E) CXCL11. (F) CCL2. (G) CXCL10. (H) TNF-α. Two-way ANOVA with multiple comparisons between all groups was used for statistical analysis for each cytokine. Data are presented as mean ± SD. P-values are presented within each time point; ∗*p* < 0.05, ∗∗*p* < 0.01, ∗∗∗*p* < 0.005, ∗∗∗∗*p* < 0.0001.

Astrocytes are known to secrete both pro- and anti-inflammatory cytokines, depending on their environment. For this reason, we decided to investigate whether the *APOE* genotype influences the astrocytes’ tau-mediated inflammatory response, using untreated astrocytes of each genotype as a control. Initially, we used a 42-target cytokine array, to compare the relative concentration of cytokines in ACM from Tau-F exposed *APOE 2/2* and *APOE 4/4* astrocytes at 3days+4days and 3days + 12days (Figure S6). Out of the 42 measured, most cytokines were released at a low level from both genotypes with a few exceptions. We noticed that ACM from *APOE 2/2* astrocytes contained higher levels of the pro-inflammatory cytokines CCL2 (MCP-1) and IL-8, compared to *APOE 4/4* astrocytes at both time points. Immunostainings revealed a similar inflammation pattern, with *APOE 2/2* astrocytes expressing higher levels of vimentin and GFAP, compared to *APOE 4/4* astrocytes (Figures 3A and 3B). However, vimentin was significantly elevated in tau-exposed *APOE 4/4* astrocytes at 3days + 12days (Figure 3A), while GFAP was not (Figure 3B). To verify these findings, we measured the cytokines in ACM from both control and Tau-F exposed *APOE 2/2* and *APOE 4/4* astrocytes over time using a custom U-Plex MesoScale format, significantly more sensitive than the cytokine array. Out of 10 targets, four cytokines (IL-1β, IL-6, IL-10, and IL-17A) were below the detection limit and thus not addressed. A comparison of the other six supported our findings that *APOE 2/2* astrocytes appear more inflammatory than *APOE 4/4* astrocytes. The cytokines CXCL11 (I-TAC), IL-12/IL23p40, IL-8, CXCL10 (IP-10) and CCL2 were all found at significantly higher concentration in *APOE 2/2* ACM, compared to *APOE 4/4* ACM (Figures 3C–3H). In particular, IL-8 (Figure 3C), CXCL10 (Figure 3G) and CCL2 (Figure 3F) were highly expressed. The concentration of IL-8 was 4x higher in *APOE 2/2* ACM, compared to *APOE 4/4* ACM at the first time point, and 6x higher at the second time point (Figure 3C). The concentrations of CXCL10 was 2-3x higher in *APOE 2/2* ACM compared to *APOE 4/4* ACM at both time points (Figure 3G). Moreover, CXCL10 release was in part affected by tau exposure. Tau-F exposed *APOE 2/2* astrocytes displayed elevated levels at 3days+4days, while *APOE 4/4* astrocytes revealed significantly higher levels at both time points (Figure 3G). CCL2, which was the most abundant cytokine (200-300x that of CXCL10) displayed the same *APOE 2/2* dominant profile (Figure 3F). Similar to CXCL10, CCL2 levels had a significant tau dependent increase, but only in *APOE 4/4* astrocytes. TNF-α was only found at low levels and remained stable irrespective of *APOE* genotype, time point, or tau exposure (Figure 3H). IL-12/IL23p40 (Figure 3D) and CXCL11 (Figure 3E) were also released in low quantities but showed an *APOE 2/2* dominant profile. Neither IL-12/IL23p40 nor CXCL11 appeared affected by tau exposure. The fact that *APOE 4/4* astrocytes proliferate more than *APOE 2/2* astrocytes (Figure S4), may affect the result. However, compensation for the cell number would only further exacerbate the difference in secreted cytokines/astrocytes between *APOE 2/2* and *APOE 4/4* astrocytes.

### Astrocytes secrete seeding competent tau aggregates irrespective of APOE genotype

**Figure 4:**
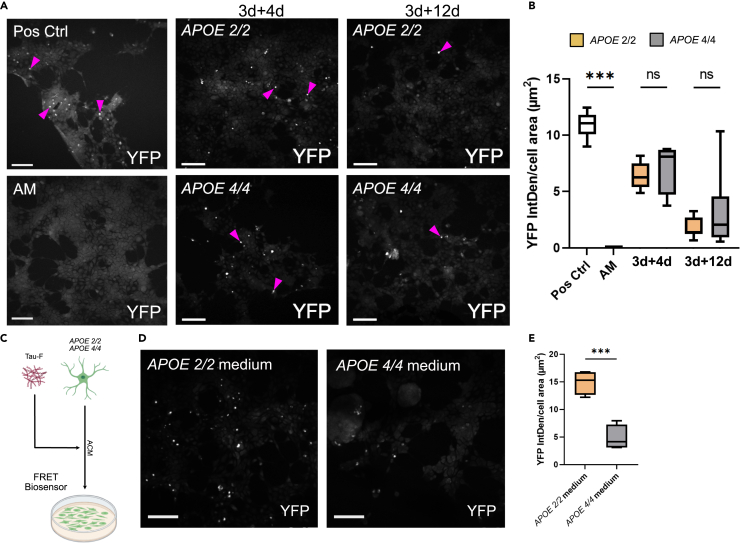
Seeding competent Tau-F aggregates are excreted from astrocytes of both genotypes but seeding is more efficient in the presence of ApoE 2 RD tau P301S FRET biosensor cells treated with ACM from Tau-F exposed APOE 2/2 or APOE 4/4 astrocytes. Positive control is the starting medium (same as the astrocytes were exposed to at day 0), and negative control is the astrocyte medium without tau (AM). All medium was fortified with 1% lipofectamine and 10% FBS. (A) Representative YFP images of Biosensor cells after 48h exposure to ACM from the first and last time point (3days+4days ACM, 3days + 12days ACM) plus positive and negative controls. The purple arrows indicate examples of positive puncta. Scale bars = 100 μm. (B) Quantification of YFP IntDen normalized to the cell area. Analyzed using one-way ANOVA with multiple comparisons between APOE 2/2 and APOE 4/4 for each time point. (C) Schematic figure of the experimental layout. Tau-F was diluted in ACM from control astrocytes and added to FRET biosensor cells. (D) Representative YFP images of Biosensor cells exposed to tau diluted in ACM from APOE 2/2 (APOE 2/2 medium) and APOE 4/4 astrocytes (APOE 4/4 medium). Scale bars = 100 μm. (E) Quantification of YFP IntDen, normalized to the cell area. Data are presented as mean ± SD. P-values are presented as following; ∗*p* < 0.05, ∗∗*p* < 0.01, ∗∗∗*p* < 0.005, ∗∗∗∗*p* < 0.0001.

Considering the substantial difference in intracellular tau deposits depending on the *APOE* genotype, we hypothesized that the *APOE 2/2* and *APOE 4/4* astrocytes may secrete various tau species, possibly with different seeding efficiencies. We have previously shown that astrocytes secrete seeding competent tau species in low concentrations over time.^20^ To determine whether the seeding efficiency of astrocyte derived tau species is affected by *APOE* genotype, we exposed the RD tau P301S FRET Biosensor cell line (HEK293T cells) to ACM from tau exposed *APOE 2/2* and *APOE 4/4* astrocytes. We observed positive FRET signal for both genotypes and at all time points, indicating that both *APOE 2/2* and *APOE 4/4* astrocytes can propagate tau pathology and do so continuously (Figure 4A). Quantification of the FRET signal indicated a roughly equivalent seeding efficiency for *APOE 2/2* and *APOE 4/4* ACM that waned with time (Figure 4B). To determine whether ApoE had a direct effect on seeding, we added Tau-F to medium containing either ApoE 2 or ApoE 4 (ACM from control *APOE 2/2* or *APOE 4/4* astrocytes, respectively) (Figure 4C). Here we noted a very distinct difference depending on the ACM source. The addition of tau to ACM from control *APOE 2/2* astrocytes resulted in a 3-fold higher YFP signal compared to ACM from control *APOE 4/4* astrocytes, suggesting that tau seeds pathology more efficiently in a medium containing ApoE 2 (Figures 4D and 4E).

### ApoE 3 influences Tau processing in *APOE 2/2* and *APOE 4/4* astrocytes

**Figure 5:**
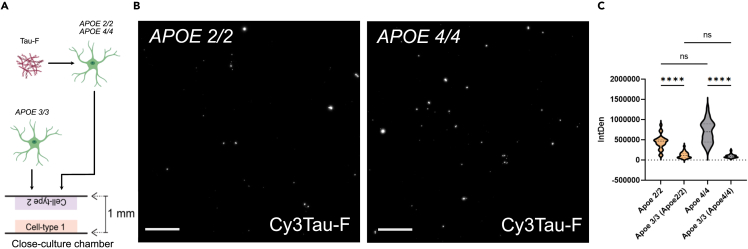
Close-culture experiments, indicate that presence of ApoE 3 influences the Tau processing in *APOE 2/2* and *APOE 4/4* astrocytes (A) Schematic outline of the experimental layout. *APOE 2/2* and *APOE 4/4* astrocytes were exposed to Tau-F before being placed in a “close-culture” chamber with *APOE 3/3* astrocytes. (B) Representative images of intracellular Cy3Tau-F signal in *APOE 2/2* and *APOE 4/4* astrocytes after four days of close-culturing (the complete images for all groups and an overview in gray-scale are shown in Figures S8 and S9). Scale bars = 100 µm. (C) Quantification of tau deposits in the astrocytes (*APOE 2/2* and *APOE 4/4* with their corresponding *APOE 3/3* astrocytes), intracellular Cy3Tau-F IntDen, normalized to the number of nuclei per field. Data are analyzed using one-way ANOVA with multiple comparisons between each group. Data are presented as mean ± SD. P-values are presented as following; ∗*p* < 0.05, ∗∗*p* < 0.01, ∗∗∗*p* < 0.005, ∗∗∗∗*p* < 0.0001.

ApoE is known to form both homo- and heterodimers.^29^ Hence, we next sought to investigate whether the presence of ApoE 3 could influence Tau processing in *APOE 2/2* and *APOE 4/4* astrocytes. For this purpose, we utilized a close-culture system. *APOE 2/2* and *APOE 4/4* astrocytes were exposed to Tau-F for three days, washed, and placed in the close culture chamber for a subsequent four days of culture together with *APOE 3/3* astrocytes (separated by a 1 mm gap) (Figures 5A and S7). Interestingly, the close cultures revealed a converse result, compared to the astrocyte mono-cultures (Figures 5B, S8, and S9). When in the proximity of *APOE 3/3* astrocytes, the *APOE 4/4* astrocytes had equivalent levels of intracellular tau deposits at 3days+4days, compared to the *APOE 2/2* astrocytes (Figure 5C). This was significantly different from what was observed in the mono-culture set up where *APOE 2/2* astrocytes displayed a much higher signal relative *APOE 4/4* at all time points (Figures 2A and 2B). We reasoned that there must be an ApoE 3 dependent effect that allows the *APOE 4/4* astrocytes to retain the ingested material.

### Astrocytic APOE-genotype does not have an immediate effect on neuronal health

**Figure 6:**
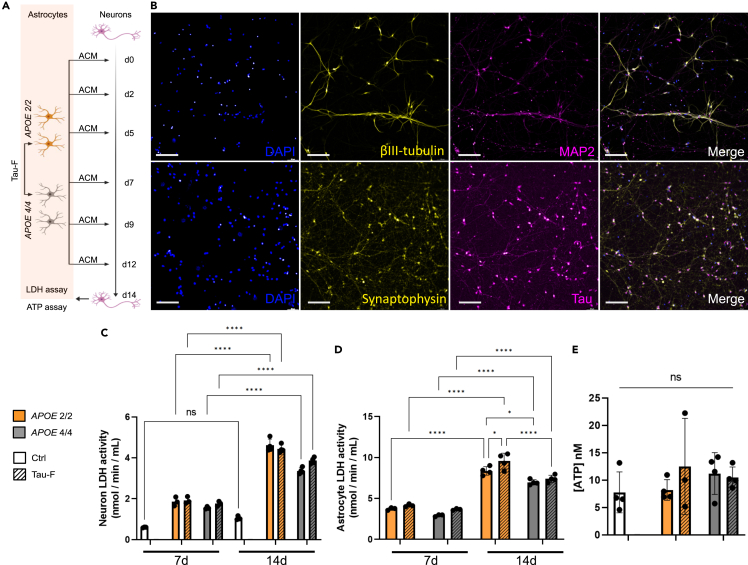
ACM from *APOE 2/2* and *APOE 4/4* astrocytes does not have an immediate effect on neuronal health (A) Schematic outline of the experimental layout. Neurons were cultured for two weeks with ACM from either *APOE 2/2* or *APOE 4/4* astrocytes (with or without tau inclusions). Addition of a regular astrocyte medium (not conditioned) was used as a control. Neuronal health was assessed using an LDH assay at d7 and d14, as well as an ATP assay at d14. (B) Example images of control neurons at day 28 of differentiation (d0 of exposure), stained for βIII-tubulin, MAP2, synaptophysin, and tau. Scale bars = 100μm. Quantification of LDH assay for (C) neurons and (D) astrocytes. Assay was performed on the medium from neurons and astrocytes at 7days and 14days (prior to medium change). Values are plotted as LDH enzymatic activity (nmol/min/mL). (E) Quantification of ATP in neurons after 14 days of exposure to ACM from *APOE 2/2* or *APOE 4/4* astrocytes, with or without tau. Regular astrocyte medium was included as a control (Ctrl). Two-way ANOVA with multiple comparisons for all mean values was used for statistical analysis. Data is presented as mean ± SD. P-values are presented as following; ∗*p* < 0.05, ∗∗*p* < 0.01, ∗∗∗*p* < 0.005, ∗∗∗∗*p* < 0.0001.

It has previously been reported that the human *APOE 4/4* genotype is linked to increased tau-mediated neuronal death in mice.^30^^,^^31^ Hence, we next sought to compare how *APOE 2/2* and *APOE 4/4* astrocytes ACM affect human neurons. For this purpose, we exposed iPSC derived neurons to ACM from control/Tau-F exposed *APOE 2/2* and *APOE 4/4* astrocytes for 14 days (Figure 6A). Immunostainings for the neuronal markers βIII-tubulin, MAP2, synaptophysin, and tau verified that the neuronal differentiation was successful (Figure 6B). To evaluate neuronal health over time, we measured LDH activity in the neuronal medium at 7 and 14 days. Although there was an increase in LDH activity over time, there was no significant difference between neurons exposed to ACM from control or Tau-F exposed astrocytes. The levels were also equivalent for neurons exposed to ACM from each genotype, indicating no apparent effect due to genotype or tau exposure (Figure 6C). Analysis of astrocyte medium at the same time points revealed a similar increase in LDH activity over time, indicating that the active LDH in the neuronal cultures likely came from the ACM and not from the neurons themselves (Figure 6D). To make sure that this effect was not due to a change in the number of neurons, we confirmed that the average number of neurons in each condition remained unchanged over time (Figure S10). Interestingly, we noted a significantly higher LDH activity in *APOE 2/2* astrocytes compared to *APOE 4/4* astrocytes at the 3days + 12days (Figure 6D). Moreover, there was also a tau dependent increase in *APOE 2/2* cultures at the later time point (Figure 6D). To clarify the effect of the ACM on neuronal health, we next analyzed ATP levels in neuronal lysates after 14 days. We did not detect any changes in ATP levels in neurons following the addition of ACM from control/Tau-F exposed *APOE 2/2* and *APOE 4/4* astrocytes (Figure 6E). Taken together, our data indicate that none of the ACMs contained neurotoxic substances at concentrations that severely affected the neurons within two weeks.

## Discussion

ApoE exists as three isoforms; *APOEε2, APOEε3* and *APOEε4.* Carrying the *APOE*ε4 allele is the strongest genetic risk factor for sporadic AD. Yet, very little is known about the role of the ApoE protein in the diseased brain and how the *APOE* genotype may affect different pathological processes on a cellular level. In contrast, *APOEε2* is associated with a decreased risk of developing AD, compared to the most common genotype *APOEε3*. However, other studies suggest that *APOEε2* instead increases the risk for other conditions, including the tauopathies PSP and AGD. Patients with these diseases display distinct astrocytic tau pathology; tufted astrocytes for PSP and granular/fuzzy astrocytes for AGD.^11^^,^^14^^,^^32^ Hence, clarifying the role of ApoE in tau pathology is central to understanding the development and progression of several neurodegenerative diseases. Astrocytes are well known as the main producers of brain ApoE. Using an *APOEε4*-to-*APOEε2* switch mouse model, (where researchers could change the expressed APOE genotype), it was shown that an astrocyte, but not a microglia-driven *APOEε4*-to-*APOEε2* switch, reduced amyloid load and improved cognition.^33^ In accord, it was recently demonstrated that a microglial specific knock-out of *APOE* did not affect the amyloid load or associated pathology.^34^ We have previously demonstrated that astrocytes are very effective at engulfing tau aggregates, but fail to degrade them. The consequence of this is large intracellular deposits of tau in astrocytes that may act as a reservoir, spreading pathological proteins to neighboring cells via tunneling nanotubes (TNTs) and secretion.^20^^,^^28^ Other research groups have shown that both astrocytes and microglia can become neurotoxic as a result of failures in the cellular degradation systems.^35^ Hence, astrocytes may act detrimentally in multiple ways during pathology, by spreading the disease and contributing to the death of surrounding neurons. In this study, we aimed to clarify in which way the *APOE* genotype influences astrocyte-mediated tau pathology, by comparing intracellular accumulation, secretion, inflammation, seeding efficiency, and neurotoxic effects posed by isogenic *APOE 2/2* and *APOE 4/4* astrocytes. However, it is important to remember that APOEε3 is the most common genotype and represents a valuable baseline reference. Follow up studies, including astrocytes homozygous for *APOEε3*, as well as heterozygous APOE 2/3 and APOE 4/3 astrocytes, would therefore be needed to fully grasp the pathological impact of ApoE.

A comparison of the intracellular tau accumulation in *APOE 2/2* and *APOE 4/4* astrocytes showed a remarkable difference. Internalized Cy3Tau-F signal was retained to a much greater extent in *APOE 2/2* astrocytes than in APOE 4/4 astrocytes. In both cases, large tau aggregates were primarily stored in aggrosomes, while some smaller aggregates were found in lysosomes. Although both genotypes experienced a decrease in intracellular tau load over time, it was substantially more prominent in *APOE 4/4* astrocytes. What this actually means for the disease progression remains to be determined, but it highlights an ApoE specific difference that could in part explain the involvement of *APOE* as a risk factor. Especially considering that the intracellular Cy3Tau-F signal could be retained in *APOE 4/4* astrocytes by culturing the cells in a “close-culture” chamber with *APOE 3/3* astrocytes. Interestingly, tau monomers have been shown to bind directly to recombinant ApoE 2 and ApoE 3 protein (strongest binding to ApoE 2), but virtually not at all to ApoE 4.^10^ However, it is worth noting that this was performed using the bare proteins in isolation. Our IP-ELISA indicates that in a more biological setting, tau-complexes are formed with both ApoE 2 and ApoE 4, even though only a small fraction of the total tau is bound to ApoE. Hence, direct protein-protein interaction does not explain the difference in tau accumulation. Another recent publication shows that human ApoE 4 reduces uptake as well as clearance in murine astrocytes.^36^ This result differs from our ELISA analysis of ACM from human astrocytes. However, while our study took place over several days, the mouse study focused on the first hours after tau exposure. In addition, mouse astrocytes and human astrocytes differ in size, function, and complexity. According to our data, three days of incubation is sufficient for human astrocytes to engulf the majority of the tau aggregates in the medium. The ingestion capacity was the same for *APOE 2/2* astrocytes and *APOE 4/4* astrocytes. We have previously shown that astrocytes not only store but also modify ingested tau aggregates, making it difficult to stain the deposits with conventional immuno-labelling.^20^ Interestingly, our western blot data on the insoluble tau fractions suggests that this process can be at least partially dependent on *APOE* genotype. Human samples display multiple bands or smears in tau-WBs due to the presence of several splice-variants and post-translational modifications (PTMs). However, in this study, we used recombinant protein exclusively of the full-length isoform (441-tau), where only one band would be expected if no modifications occurred within the cells. Interestingly, the higher molecular weight tau, exclusively found in *APOE 2/2* astrocytes, is similar to that reported for paired helical filaments (PHFs).^37^^,^^38^ This difference in the insoluble fraction also fits well with the overall pattern seen by microscopy of the Cy3Tau-F. Taken together, we reason that the main effects posed by *APOE* is a significant storage difference rather than an uptake difference, since the levels in the medium and the soluble fraction of WBs were similar. Moreover, the two distinct bands in the insoluble fraction suggest an *APOE 2/2* dominant difference in the cellular processing and modification of the ingested tau.

Inflammatory pathways have been suggested as potential contributors to the propagation of tau pathology.^39^ However, to date most studies have focused on microglia. Astrocytes are known to be a central player in neuroinflammation and produce both pro- and anti-inflammatory cytokines depending on the situation.^40^ Moreover, astrocytes have the capacity to act as antigen-presenting cells.^41^ In our initial experiment, using a 42-target array, we noticed that both *APOE 2/2* and *APOE 4/4* astrocytes excrete many cytokines, but mostly in low concentrations. IL-8 and CCL2 were the main exceptions, excreted at much higher concentrations. Interestingly, *APOE 2/2* ACM contained increased levels of these cytokines compared to *APOE 4/4* ACM at both time points. To be able to assess the differences in cytokine production between the two genotypes more accurately we performed MSD-ECL analysis. Based on our initial results and previous reports, we decided to measure the ACM levels of IL-1β, IL-6, IL-8, IL-10, IL-12/IL-23p40, IL-17A, CXCL11, CXCL10, CCL2 and TNF-α. These include both pro- and anti-inflammatory molecules that all work at different levels of inflammatory pathways. In line with our initial experiment, IL-8 and CCL2 were highly expressed, with significantly higher levels in *APOE 2/2* ACM, compared to *APOE 4/4* ACM. Both IL-8 and CCL2 are chemoattractant agents that are involved in monocyte/macrophage infiltration. IL-8 has been shown to facilitate tau phosphorylation and is generally elevated in cerebral spinal fluid of patients with AD.^42^^,^^43^ Moreover, IL-8 gene polymorphisms have been shown to increase the risk of developing sporadic AD, suggesting an inflammation driven predisposition.^44^ Additionally, CCL2 is increased in the plasma of patients with AD.^45^ A similar trend for both IL-8 and CCL2 have been reported in human CSF, although the authors themselves note a limiting sample size.^46^ The chemokine CXCL10, was also significantly higher in *APOE 2/2* ACM than *APOE 4/4* ACM. CXCL10 is generally produced as a response to IFN-γ signaling and promotes monocyte/macrophage infiltration as well as angiogenesis. Overall, the cytokine response appears to be much more robust in *APOE 2/2* astrocytes, compared with *APOE 4/4* astrocytes within the two week period. CCL2 and CXCL10 were significantly increased in tau exposure *APOE 4/4* astrocytes for all time points. This was conversely not the case for *APOE 2/2* astrocytes, where the only difference observed was at an early time point for CXCL10. As a whole, our data suggest that the *APOE 2/2* genotype results in a significantly stronger inflammatory response than the *APOE 4/4* genotype, at least in the acute stage. This was an unexpected result, and future investigations, including *APOE 3/3* astrocytes will give important information about the *2/2* and *4/4* astrocytes' inflammation status, in relation to the base line. However, the main driver for cytokine production was genotype and tau exposure only seemed to contribute moderately. This is in line with our precious study where cytokine release by astrocytes as well as microglia seemed unaffected by α-synuclein and Aβ exposure.^23^

We have previously demonstrated that astrocytes excrete seeding competent tau species.^20^ Here, we were interested in whether the *APOE* genotype could affect the seeding efficiency of the excreted tau species. Comparing the FRET signal emitted from biosensor cells exposed to ACM from *APOE 2/2* and *APOE 4/4* astrocytes showed no significant difference. Hence, we concluded that the modifications we observed in the insoluble fraction of *APOE 2/2* astrocytes do not influence the seeding efficiency of excreted tau species in any significant way. Interestingly, we observed that unmodified Tau-F seed was more efficient in a medium containing ApoE 2. Since we did not observe any isoform dependent differences in protein-protein binding between tau and ApoE, this likely occurs indirectly through a different mechanism. Taken together, we reason that ApoE 2 can lead to more seeding over all, by increasing the cell-to-cell delivery of toxic tau aggregates, rather than by the creation of more aggregation prone tau species.

We have previously reported that neurons in co-culture with tau burdened astrocytes had a reduced frequency in spontaneous excitatory postsynaptic currents (sEPSC).^20^ However, ACM from *APOE 2/2* or *APOE 4/4* astrocytes had no clear impact on neuronal health as measured by changes LDH activity and ATP levels. Many studies have linked *APOEε4* to greater neuron loss, both in mice and organoids.^30^^,^^31^^,^^47^ With that in mind, it is possible that the two weeks of incubation in our experiments are insufficient for significant neuronal damage to occur.

For several tauopathies, including AD, the *APOE* genotype is the major genetic risk factor. However, the underlying mechanisms, including the link between ApoE and astrocyte-mediated tau pathology are still unclear. In conclusion, our data show that the *APOEε2* and *APOEε4* genotypes differ significantly when it comes to tau accumulation and tau modification in human astrocytes. However, there was no distinction in seeding efficiency or neurotoxicity between the two genotypes, at least not in the short term. Moreover, *APOEε2* astrocytes show a substantial inflammatory response, compared to *APOEε4* astrocytes. It is important to note that the biological significance of the presented results on *APOE 2/2* or *APOE 4/4* astrocytes would depend on how the data relate to *APOE 3/3* astrocytes. Without the *APOEε3* cells, we can only draw conclusions regarding the *APOEε2 and APOEε4* interrelationship, but not be sure if their relative status differs from the most common genotype. In order to identify novel treatment targets for AD and other tauopathies, detailed knowledge about the mechanisms by which ApoE influences tau pathology is highly desirable. Taken together, our results highlight the importance of astrocytes in this context.

### Limitations of the study

In this study, we compare tau accumulation, tau spreading, tau-mediated inflammation and neurodegeneration in isogenic human iPSC derived astrocytes, homozygous for *APOEε2* and *APOEε4*. The experimental set-up was based on the fact that *APOEε4* is the greatest genetic risk factor for developing AD, while *APOEε2* is a significant risk factor for developing other diseases, including PSP, AGD, and CAA. The study was originally designed to include *APOEε3/3* astrocytes in all experiments. However, despite multiple attempts of differentiation, the purchased isogenic *APOEε3/3* iPSC*-*line failed to produce functional astrocytes, due to poor cell viability. For this reason, we were unable to include the ε3 variant in our comparisons, which limits the overall scope of the study. Globally, the prevalence of ε2, ε3, and ε4 alleles is approximately 7, 79 and 14%, respectively. Hence, the most common forms are APOE 3/3, APOE 2/3, and APOE 4/3, and *APOE*ε3 is considered a “base-line” for ApoE functionality. To understand the biological significance of astrocytic ApoE in depth and to fully explore the effects of *APOE* genotype on astrocytic tau processing, it would be necessary to include APOE 3/3 astrocytes in future studies. Another limitation of the study is the low number of cell lines and follow-up investigations would benefit from including several parallel sets of isogenic cell lines.

## Resource availability

### Lead contact

Further information and requests for resources and reagents should be directed to and will be fulfilled by the Lead Contact, Anna Erlandsson (anna.erlandsson@pubcare.uu.se).

### Materials availability

This study did not generate new unique reagents. Further information and requests for resources and reagents should be directed to and will be fulfilled by the lead contact, Anna Erlandsson (anna.erlandsson@pubcare.uu.se).

### Data and code availability

Data: All data generated is included in the article and supplementary figures.

Code: In this study we used custom macros for quantification. Further information and requests of macro details should be directed to and will be fulfilled by the lead contact, Anna Erlandsson (anna.erlandsson@pubcare.uu.se).

Other items: Any requests for additional information should be directed to the lead contact, Anna Erlandsson (anna.erlandsson@pubcare.uu.se).

## Acknowledgments

We thank the iPS Core at Karolinska Institutet for providing the Cntr9 NES-cells, the BioVis core facility at Uppsala University for technical assistance with TEM, Uppsala University’s 3D-printing facility (U-PRINT) for producing and designing the "close-chamber" and the team at SciLifeLab Affinity Proteomics for helping with the MSD-ECL analysis.

This study was supported by grants from the 10.13039/501100004359Swedish Research Council (2021-02563), the Swedish Alzheimer Foundation (AF-980656), 10.13039/501100005701Åhlén Foundation (233044), the 10.13039/501100003792Swedish Brain Foundation (FO2022-0083), Olle Engkvist Byggmästare Foundation (215-0399), Stohnes Foundation (2023), Sävstaholms Foundation (ST-2023-022) and the 10.13039/100009487Swedish Fund for Research without Animal Experiments (F2022-0004).

## Author contributions

TM designed the study, optimized and performed experiments, interpreted data, and wrote the article; EK developed cell culture protocols, performed DNA extractions, and revised the article; KE performed experiments, interpreted data, and revised the article; AD cultured cells, developed culture protocols, and revised the article; JR interpreted data and revised the article; AE designed the study, interpreted data, coordinated the study, secured the funding and wrote the article. All authors have read and approved the final article.

## Declaration of interests

The authors declare that no competing interests.

## STAR★Methods

### Key resources table

**Table:** 

REAGENT or RESOURCE	SOURCE	IDENTIFIER
**Antibodies**		

T46	Thermo Fisher Scientific	13-6400
Tau-5	Thermo Fisher Scientific	MA5-12808
BT2	Thermo Fisher Scientific	MN1010
BT2-biotinylated	Thermo Fisher Scientific	MN1010-B
Tau-12	Thermo Fisher Scientific	806501
Anti-Vimentin	Sigma Aldrich	ab5733
Anti-GFAP	Abcam	ab4674
YE269	Abcam	ab32127
TUBB3	Nordic Biosite	801202
BioTracker 490	Sigma Aldrich	SCT106
16H22L18	Thermo Fisher Scientific	701241
NBP1-87102	Novusbio	NBP1-87102
Goat Anti-Mouse ALEXA	Thermo Fisher Scientific	A11029
Goat Anti-Rabbit ALEXA	Thermo Fisher Scientific	A11008
Goat Anti-Chicken ALEXA	Thermo Fisher Scientific	A11039
Goat Anti-Mouse DyLight	Invitrogen	SA525521
Goat Anti-Rabbit DyLight	Invitrogen	35568

**Chemicals, peptides, and recombinant proteins**		

Advanced DMEM/F12	Thermo Fisher Scientific	12634-010
Penicillin-Streptomycin	Thermo Fisher Scientific	15140-122
L-Glutamine	Thermo Fisher Scientific	25030-024
B27	Thermo Fisher Scientific	11530536
non-essential amino acids	Thermo Fisher Scientific	11140050
bFGF	Thermo Fisher Scientific	13256029
Heregulin beta-1	Sigma Aldrich	SRP3055
Activin A	Peprotech	120-14E
IGF-1	Sigma Aldrich	SRP3069
CNTF	Thermo Fisher Scientific	PHC7015
Poly-L-Ornithine	Sigma Aldrich	P3655
Laminin	Sigma Aldrich	L2020
Trypsin-EDTA	Thermo Fisher Scientific	10779413
DMEM/F12+Glutamax	Thermo Fisher Scientific	31331028
N2 Supplement	Thermo Fisher Scientific	11520536
Neurobasal medium	Thermo Fisher Scientific	21103049
GlutaMAX	Thermo Fisher Scientific	35050038
Recombinant human 441-tau	Anaspec	AS-55556
MES Hydrate	Sigma Aldrich	M2933
1,4-Dithiotheitol	Sigma Aldrich	D0632
Heparin	Sigma Aldrich	H3149
Paraformaldehyde	Sigma Aldrich	158127
Normal Goat Serum	Bio Nordika	S-1000-20
Triton-X	Sigma Aldrich	T8787
EverBrite Hardset (+DAPI)	VWR	23004
EverBrite Hardset	VWR	23003
Halt Protease Inhibitor Cocktail	Thermo Fisher Scientific	78430
Bolt sample reducing agent	Invitrogen	B00009
LDS sample buffer	Invitrogen	NP0007
4-12% Bis-Tris Plus Gel	Invitrogen	NW04125BOX
PageRuler Plus	Thermo Fisher Scientific	26619
MES SDS running buffer	Thermo Fisher Scientific	B0002
Bovine Serum Albumin	Sigma Aldrich	A7030-100G
Aqueous TMB substrate	Lumiradx	331177
H_2_SO_4_	Sigma Aldrich	7664-93-9
Lipofectamine 3000	Thermo Fisher Scientific	L3000015
Fetal Bovine Serum	Thermo Fisher Scientific	11533387

**Critical commercial assays**		

42-target cytokine array	Abcam	ab133997
Colorimetric LDH assay	Abcam	ab102526
Luminescent ATP assay	Abcam	ab113849
PureLink genomic DNA kit	Invitrogen	K182001
Cy3-labeling kit	Amersham	PA33000
Pierce BCA protein assay	Thermo Fisher Scientific	23225
no-stain Protein Labeling Reagent	Thermo Fisher Scientific	A44449

**Experimental models: Cell lines**		

BIONi037-A-2	Sigma Aldrich	BIONi037-A-2
BIONi037-A-4	Sigma Aldrich	BIONi037-A-4
Cntrl-9 II	Karolinska Institutet	Cntrl-9 II
Tau RD P301S Biosensor	ATCC	CRL-3275

**Software and algorithms**		

Zen 3.1 (blue edition)	Zeiss	https://www.micro-shop.zeiss.com/en/de/softwarefinder/software-categories/zen-blue/
Zen (black edition)	Zeiss	https://www.micro-shop.zeiss.com/en/de/softwarefinder/software-categories/zen-black/
LAS X	Leica	https://www.leica-microsystems.com/products/microscope-software/p/leica-las-x-ls/
Image Lab 6.1	Bio-Rad Laboratories Inc	https://www.bio-rad.com/en-se/product/image-lab
ImageStudie	LI-COR Biosciences	https://www.licor.com/bio/image-studio/
Magellan	Tecan	https://lifesciences.tecan.com/plate_readers/infinite_200_pro?p=tab--3
FIJI (ImageJ)	Open Access	https://imagej.net/software/fiji/downloads
GraphPad Prism 10	GraphPad Software	https://www.graphpad.com/resources

### Experimental model and study participant details

#### Ethics approval and consent to participate

Not applicable.

#### Cell lines

Human isogenic *APOE 2/2* and *APOE 4/4* astrocytes were generated from neuroepithelial-like stem (NES) cells, produced from the human induced pluripotent stem cell (hiPSC) lines: EBiSC BIONi037-A-2 = APOE-ε2/ε2 (Merck) and EBiSC BIONi037-A-4 = APOE-ε4/ε4 (Merck). Cells were cultured in DMEM/F12 based medium (details below) under standard culturing conditions of 37°C, 5% CO_2_. The *APOE 3/3* line (iPSCs, Cntrl9 II) used for the close culture experiments (described below) was cultured in the same way.^21^ APOE homozygosity was determined using PureLink DNA assay according to the manufacturers instructions.

The tau RD P301S Biosensor HEK cell line (ATCC, CRL-3275) was cultured in DMEM based medium fortified with 10% FBS at 37°C and 5% CO_2_. All cell lines were cultured with 1% penicillin-streptomycin.

### Method details

#### Culturing of human iPSC-derived astrocytes

To generate astrocytes, NES cells were cultured in Advanced DMEM/F12 (Thermo Fisher, 12634-010) supplemented with 1% penicillin-streptomycin (Thermo Fisher, 15140-122), 1% L-glutamine (Thermo Fisher, 25030-024), 1x B27 (Thermo Fisher, 11530536) and 1x non-essential amino acids (Thermo Fisher, 11140050). The following factors were added to the medium right before use: 8 ng/ml bFGF (Thermo Fisher, 13256029), 10 ng/ml heregulin beta-1 (Sigma, SRP3055), 10 ng/ml activing A (Peprotech, 120-14E), 200 ng/ml IGF-1 (Sigma, SRP3069). From week three of differentiation, 20 ng/ml of CNTF (Thermo Fisher, PHC7015) was also included. A full medium change was performed every other day for the duration of the differentiation. Cells were cultured in cell culture flasks (Sarstedt) coated with 100 μg/ml poly-L-ornithine (Sigma, P3655) and 50 μg/ml laminin (Sigma, L2020), and seeded for experiments at 5 000 cells/cm^2^. 4% Trypsin-EDTA (Thermo Scientific, 10779413) was used for passaging the cells and the cells were differentiated for 28 days, prior to the start of experiments.

#### Culturing of human iPSC-derived neurons

Human neurons were generated from NES-cells (Cntrl9 II cell line) using a well-established protocol.^25^^,^^48^ Shortly, the NES cells were plated at 40 000 cells/cm^2^ in cell culture flasks (Sarstedt) coated with 100 μg/ml poly-L-ornithine (Sigma, P3655) and 50 μg/ml laminin (Sigma, L2020) and cultured for five days in neuronal differentiation medium: DMEM/F12+Glutamax (Fisher Scientific, 31331028) supplemented with 1% N2 (Fisher Scientific, 11520536), 1% penicillin-streptomycin (Thermo Fisher, 11548876), 1x B27 (Thermo Fisher, 17504044). During this period, the medium was fully replaced every other day. The cells were detached from the flask using 1x TrypLE (Thermo Fisher, 12563029), re-plated for experiments at a density of 20 000 cells/cm^2^ (coating was preformed like before but with 5x laminin concentration) and cultured for another five days (until d10). During this period, half of the medium was replaced every other day. From day ten and onwards the neuronal differentiation medium was mixed 1:1 with complete neurobasal medium, consisting of Neurobasal medium (Thermo Fisher, 21103049) supplemented with 1% penicillin-streptomycin (Thermo Fisher, 11548876), 1x B27 and 1x GlutaMAX (Thermo Fisher, 35050038). The neurons were differentiated for 28 days prior to experiments.

#### DNA extraction and sequencing of iPSCs

DNA was extracted from EBiSC BIONi037-A-2 and EBiSC BIONi037-A-4 cells using PureLink genomic DNA kit (Invitrogen, K182001) according to the manufacturer’s specifications. Sanger sequencing was performed by Eurofins genomics, Uppsala, Sweden.

#### Production of *in vitro* tau aggregates

Human tau fibrils (Tau-F) were generated using recombinant human 441-tau monomers (Anaspec, AS-55556). Monomers were initially dissolved (3 mg/ml) in 100 mM MES hydrate (Sigma, M2933) buffer, pH 6.5, containing 10 μM of 1,4-Dithiotheitol (Sigma, D0632) and 16.25 μM heparin (Sigma H3149) and incubated on slow shake at 37°C for 7 days. Tau aggregates were then centrifuged at 20879xg for 30 min, 4°C and the pellets re-suspended in Phosphate-buffered saline, PBS (1 mg/ml). When required, the fibrils were labelled using Amersham Cy3-labeling kit (Amersham, PA33000) according to the manufacturer’s instructions. Fibrils were used immediately or stored at -70°C. Prior to experiment, the fibrils were sonicated at 20% amplitude, 1s on/off for 30 s, using a Sonics Vibra Cell sonicator.

#### Tau exposure of astrocyte cultures

Astrocytes were exposed to 200 nM sonicated Tau-F (Cy3-labeled or unlabeled) for 3 days before being washed and cultured further in tau free medium for another 4-12 days (3d+4d, 3d+8d and 3d+12d) after which medium was collected and the cells were fixed or lysed. For neuronal exposure experiment, astrocytes were incubated with tau and cultured for 3d+2d before the medium collection started.

#### Immunocytochemistry (ICC)

Cells were fixed with 4% paraformaldehyde (Sigma) in PBS for 15 min at RT and washed twice with PBS. Blocking and permeabilization were performed by incubation in 5% normal goat serum (NGS) and 0.1% Triton X-100 in PBS for 30 min at RT. Then, the cells were incubated with primary antibodies (diluted in 0.5% NGS 0.1% Triton X-100 in PBS) overnight at 4°C, washed 3x10 min with PBS and incubated with secondary antibodies (diluted in 0.5% NGS and 0.1% Triton X-100 in PBS) for 1 h at 37°C. AlexaFluor goat-anti mouse, rabbit or chicken; 488, 555 or 647 (1:200, Molecular Probes) were used as secondary antibodies. Cells were washed 3x5 min with 1x PBS and mounted on microscope slides using Ever Brite Hardset Mounting medium with or without DAPI (VWR, 23004 and 23003). All antibodies used are listed in Table of antibodies.

#### Western blot (WB)

For lysis, cells were incubated in ice cold lysis buffer (20 mM Tris pH 7.5, 0.5% Triton X-100, 0.5% Deoxycholic acid, 150 mM NaCl, 10 mM EDTA, 30 mM Na4O7P2, supplemented with 1x Halt Protease Inhibitor Cocktail (Thermo Scientific, 78430)) for 10 min before being scraped off the plate with a cell scraper (Thermo Fisher, 99002). The cell samples were transferred to LO-bind tubes and incubated on ice for 30 min before being centrifuged at 28 000 g for 30 min (4°C). The lysates were separated from pellets and stored in -70°C until use. Pellets were diluted in equivalent amount of lysate buffer and fortified with 2% SDS and sonicated for 1 min, 2 sec ON/1 sec off at 25% AMP. Total protein concentrations of cell lysates were determined using Pierce BCA protein assay kit (Thermo Scientific, 23225) according to the manufacturer’s instructions. Protein samples (18 μg) were denatured by incubating with Bolt sample reducing agent (Invitrogen, B00009) in LDS sample buffer (Invitrogen, NP0007) for 5 min at 95°C. The samples were then loaded on a 4-12% Bis-Tris Plus Gel (Invitrogen, NW04125BOX) with 5 μl PageRuler Plus (Thermo, 26619) protein ladder and run for 20 min at 200 V in MES SDS running buffer (Thermo Fisher, B0002). Transfer was done using the Invitrogen Power Blotter (PB0010) onto mini PVDF transfer stacks (Thermo Fisher, PB5240) using mixed ranged settings. The total protein in each lane was measured by the no-stain Protein Labeling Reagent (Thermo Fisher, A44449) followed by BIO-RAD ChemiDoc XRS+ reading. Blocking was performed with 5% bovine serum albumin (BSA) in Tris Buffered Saline-Tween20 (TBS-T) for 1h at room temperature (RT). The membrane was then incubated with primary antibodies diluted in 5% BSA TBS-T at 4°C overnight and secondary antibodies (goat anti-rabbit and anti-mouse DyLight 680, and goat anti-rabbit and anti-mouse DyLight 800, diluted 1:20000 in 5% BSA, TBS-T) for 1h at RT. The signal was analyzed using an SA Odyssey (LI-COR). Band intensity was measured using the ImageStudio (LI-COR) or ImageLab (BIO-RAD) software. Each band was normalized against the lane intensity of the total protein labelling signal. Each experiment was repeated three times. All antibodies used are listed in Table of antibodies.

#### Immunoprecipitation (IP)

Invitrogen Dynabeads M-280 (Thermo, 11203D) were coupled to ApoE antibodies (Thermo, 701241, 2 mg Ab/mL). For IP, the anti-ApoE-beads were added to ACM (83.7 μL anti-ApoE-beads/mL medium) and incubated for 24 h at 4°C on shake. After thorough washing, the antigen was stripped from the beads by suspending in 200 μL 0.5% SDS (20% of the original sample volume to increase concentration to 5x) and boiled for 5 min at 95°C.

#### Sandwich ELISA

Corning high-binding 96-well plates (VWR, 734-1624) were coated with 0.5 μg/mL of T46 for 24 h at 4°C and blocked with 1% BSA for 2 h at RT. The conditioned media/lysate samples were fortified with 0.5% SDS and heated to 95°C for 5 min prior to loading onto the plates. The plates were incubated for 24 h at 4°C. Then, 0.5 μg/mL BT2-Biotin was added and the plates were incubated for another 2h at RT on mild shake. Aqueous TMB substrate (Lumiradx, 331177) was added and the plates were incubated for ∼10 min before the reaction was halted with 1 M H_2_SO_4_. Detection was performed using the Infinite M200 pro ELISA reader. Each experiment was repeated three times. All antibodies used are listed in Table of antibodies.

**Table:** Table of antibodies

Antibody/Dye	Manufacturer	Target	Method
T46	Thermo (13-6400)	Tau (404-441 aa)	WB/ELISA
Tau-5	Thermo (MA5-12808)	Tau (218-225 aa)	WB/ICC
BT2	Thermo (MN1010)	Tau (194-198 aa)	WB
BT2 (biotin)	Thermo (MN1010-B)	Tau (194-198 aa)	ELISA
Tau-12	Thermo (806501)	Tau (6-18 aa)	WB
Anti-vimentin	Sigma (ab5733)	Vimentin	ICC/WB
Anti-GFAP	Abcam (ab4674)	GFAP	ICC/WB
YE269	Abcam (ab32127)	Synaptophysin	ICC
TUBB3	Nordic Biosite (801202)	βIII-tubulin	ICC
BioTracker 490	Sigma (SCT106)	Membranes	ICC
16H22L18	Thermo (701241)	ApoE (240-251 aa)	IP/WB
NBP1-87102	Novusbio (NBP1-87102)	S100β	WB

Antibodies used in the study including epitopes and specific methods used.

#### 42-Target cytokine array

The human cytokine antibody array (abcam, ab133997) was performed according to manufacturer’s recommendations (1 mL ACM/membrane). Membranes were developed and read using a BIO-RAD ChemiDoc XRS+. Multiple images of varying exposure time were captured in order to enable reading of all dots on the membrane.

#### U-Plex MSD-ECL (MesoScale)

The MSD-ECL assay and analysis were performed with a MESO SECTOR S 600MM at SciLifeLab Affinity Proteomics, Uppsala University. Concentrations of pro-/anti-inflammatory cytokines in the medium was quantified using a costum U-Plex MesoScale format, detecting IL-1β, IL-6, IL-8, IL-10, IL-12/IL-23p40, IL-17A, CXCL11 (I-TAC), CXCL10 (IP-10), CCL2 (MCP-1) and TNF-α. Three wells per condition were included in the analysis.

#### Tau FRET seeding assay

The tau RD P301S Biosensor HEK cell line (ATCC, CRL-3275) was used to evaluate seeding efficiency of Tau-F excreted from the astrocytes. The HEK cells were cultured according to the ATCC’s recommendations. The cells were plated at 50 000 cells/cm^2^ in 24 well plates. ACM was fortified with 1% lipofectamine 3000 (Fisher Scientific, L3000015) and 10% fetal bovine serum (FBS) (Fischer Scientific, 11533387) and added to the biosensor cells for 48 hours before fixation with 4% paraformaldehyde. FRET signal (YFP emission resulting of CFP excitation) was captured using a LSM700 confocal microscope. Negative control (medium from control astrocytes) and positive control (Tau-F in medium) were both fortified with lipofectamine and FBS as described above.

#### “Close-culture” chamber system

*APOE 2/2* and *APOE 4/4* astrocytes were cultured separately on 18 mm diameter coverslips and incubated with Tau-F for three days as described above. The cells were washed with PBS before being moved to the “close-culture” chamber system^23^ together with *APOE 3/3* astrocytes (derived from the iPSC, Cntrl9 II line). The *APOE 3/3* astrocytes were cultured in parallel on 24 mm diameter coverslips. Cells were fixed after four days in the culture system (3d+4d).

#### ACM exposure of iPSC derived neurons

ACM from *APOE 2/2* and *APOE 4/4* astrocytes (ctrl and tau exposed) was added to neuronal cultures from d28 to d42. Each time, half of the medium was replaced, constituting of 1:1 ACM and standard neuronal medium (DMEM/Neurobasal). The ACM was collected just prior to the neuronal exposure. Control neurons received fresh astrocyte medium instead of ACM. Medium was changed at d0, d2, d5, d7, d9 and d12. Cultures were kept for a total of two weeks.

#### LDH assay

Colorimetric lactate dehydrogenase (LDH) assay kit (Abcam, ab102526) was used according the manufacturer’s instructions. Medium from neurons exposed to ACM (see paragraph above) was collected at d7 and d14 for analysis. ACM from the astrocyte cultures was also collected for analysis. Detection was performed with an Infinite M200 pro ELISA reader.

#### ATP assay

Neurons were cultured in a 96-well plate and exposed to ACM for 14 d (see paragraph above). Luminescent ATP detection assay kit (Abcam, ab113849) was used according the manufacturer’s instructions. Detection was performed with an Infinite M200 pro ELISA reader.

### Quantification and statistical analysis

#### Image analysis

Fluorescent images used for quantification of Cy3Tau-F, vimentin and GFAP intensities were captured with the Observer Z1 Zeiss microscope (40x objective). For the biosensor FRET quantification (YFP emission due to 405 nm laser excitation) images were captured using a LSM700 confocal microscope. All images of the neurons were captured with the Leica THUNDER imager. The z-stack images (25-30 images/z-stack) were compiled as a composite for max intensity. Each experiment was repeated three times and 12-15 randomly captured images were analysed per experiment. All quantifications were performed in ImageJ.

For the Cy3Tau-F inclusions, we developed a specific ImageJ macro. First, the region of interest (ROI) was determined (based on a cellular marker) to only include intracellular Cy3 in the measurement. Next, the corresponding Cy3 images were analysed based on the following steps: set scale, convert to 16-bit, subtract background, set threshold (the same threshold was used for all time-points), clear outside (everything outside of the ROI), set measurements and analyse particles. In each image, Cy3tau deposits were assessed by measuring the total area, the number of particles and the sum of the integrated densities (area x mean intensity of each Cy3tau deposit), normalized to the number of living cells (identified by DAPI staining).

Quantification of the biosensor YFP IntDen was performed in the same way as described for Cy3 signal. YFP IntDen was normalized to the total cell area/image (instead of DAPI since it would interfere with the CFP/YFP emission). Cell area was manually drawn for each image and measured. To allow blinding, this process was performed on corresponding phase contrast images.

#### Statistical analyses

All statistical analyses was performed in Graphpad Prism (v.9.3.1). The data sets were initially analysed using the D’Agostino-Pearson omnibus and the Shapiro-Wilk normality tests prior further analysis. Datasets that passed both normality tests were analysed using either a one-way or two-way ANOVA for datasets containing more than one group with multiple comparisons for mean values for each group and Šidák multiple analysis correction. Alternatively, datasets containing only two groups were analysed using standard t-test. Each experiment was conducted three times. P-values are presented as following; ∗ p<0.05, ∗∗ p<0.01, ∗∗∗ p<0.005, ∗∗∗∗ p<0.0001.
