# Supplementary material for: Tau processing and tau-mediated inflammation differ in human APOEε2 and APOEε4 astrocytes
Source: iScience. 2024 Oct 11;27(11):111163. doi: 10.1016/j.isci.2024.111163 (PMC11549983; doi:10.1016/j.isci.2024.111163)
Supplement: Document S1. Figures S1–S10 [file mmc1.pdf]

iScience, Volume *27*

Supplemental information

Tau processing and tau-mediated inflammation differ in human *APOEε2* and *APOEε4* astrocytes

Tobias Mothes, Evangelos Konstantinidis, Khalid Eltom, Abdulkhalek Dakhel, Jinar Rostami, and Anna Erlandsson

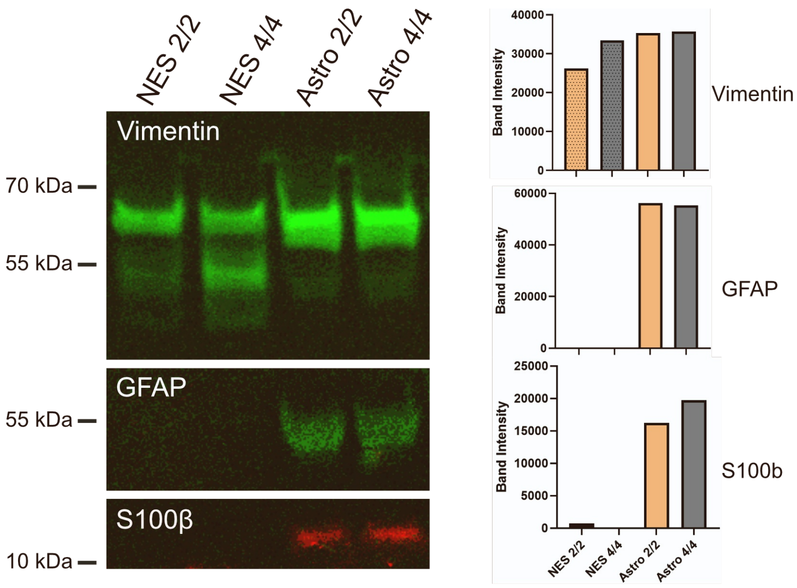

Figure S1. **Western blot characterisation of cell lines. Related to figure 1.** Western blot of cell lysates before and after astrocyte differentiation (NES cells and astrocytes).

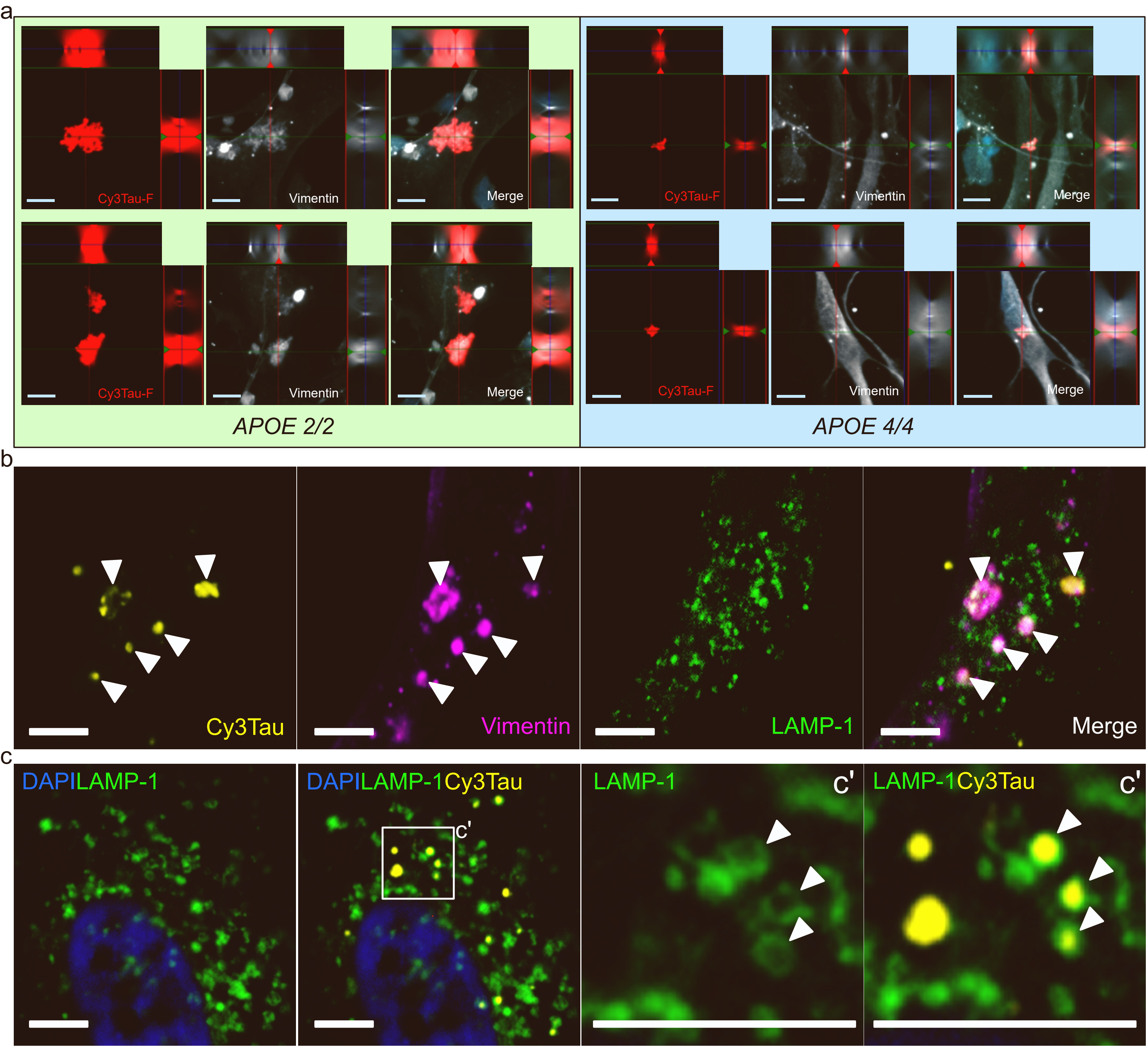

Figure S2. Localisation of internalised Cy3Tau aggregates in astrocytes. Related to Figure 1. (**a**) Tau co-localises with vimentin in *APOE 2/2* and *APOE 4/4* astrocytes. (**b**) Majority of internalized tau is stored in aggrosomes (indicated by white arrows). (**c**) Some small tau inclusions are located inside lysosomes (indicated by the white arrows). Scale bars = 10 µm.

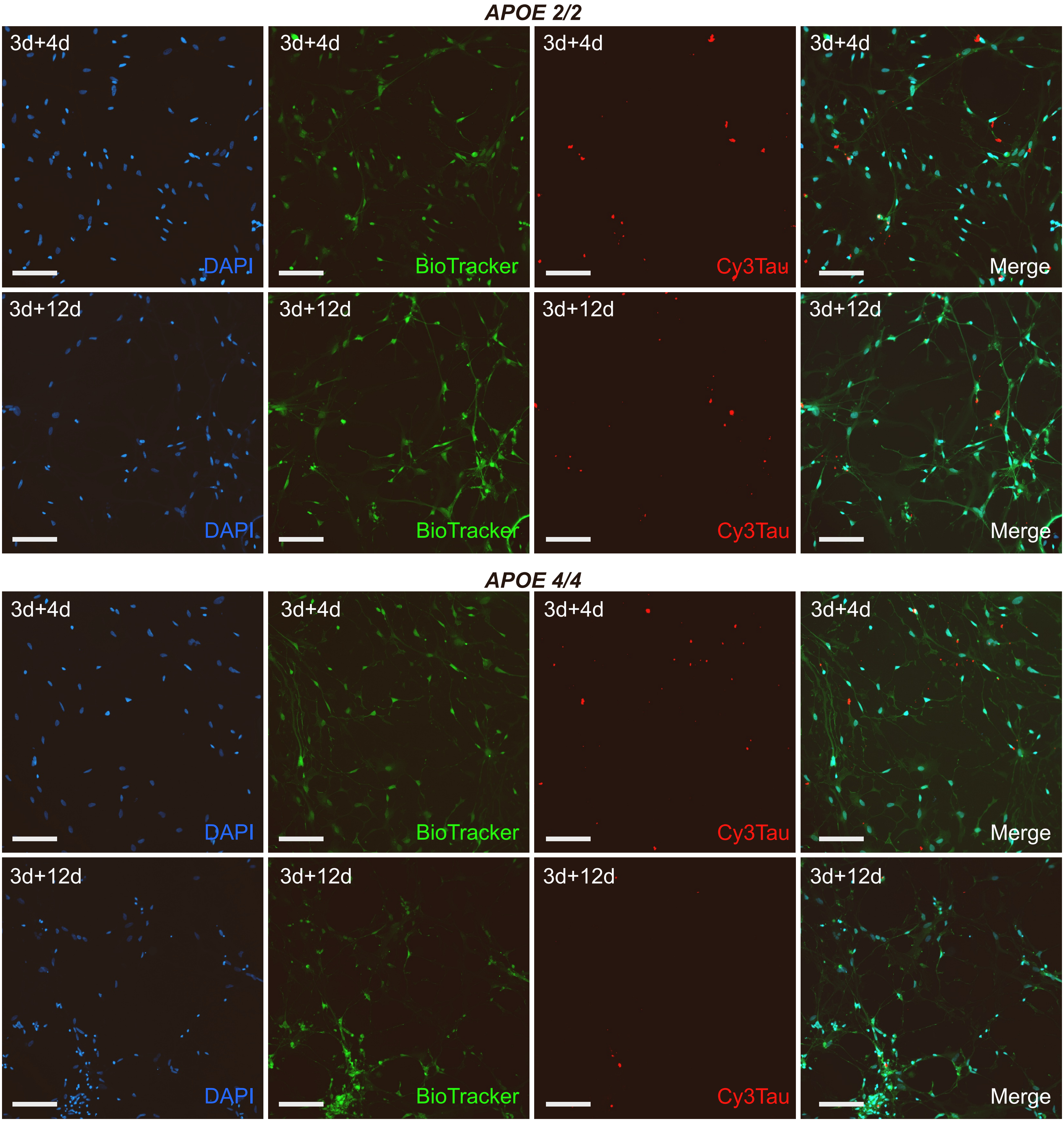

Figure S3. Complete fluorescent images of Cy3Tau in astrocytes. Related to Figure 2 a-e. Cy3Tau exposed *APOE 2/2* and *APOE 4/4* astrocytes at 3d+4d and 3d+12d. Cells were stained for DAPI and BioTracker488 (membrane dye). Scale bars = 100 µm.

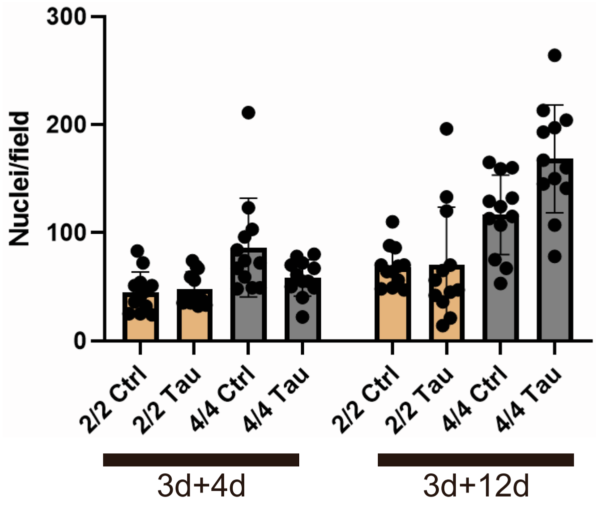

Figure S4. Cell count (astrocytes)/field of view. Related to Figure 2 a-e and Figure 3. Number of nuclei/field over time in cultures of *APOE 2/2* and *APOE 4/4* astrocytes.

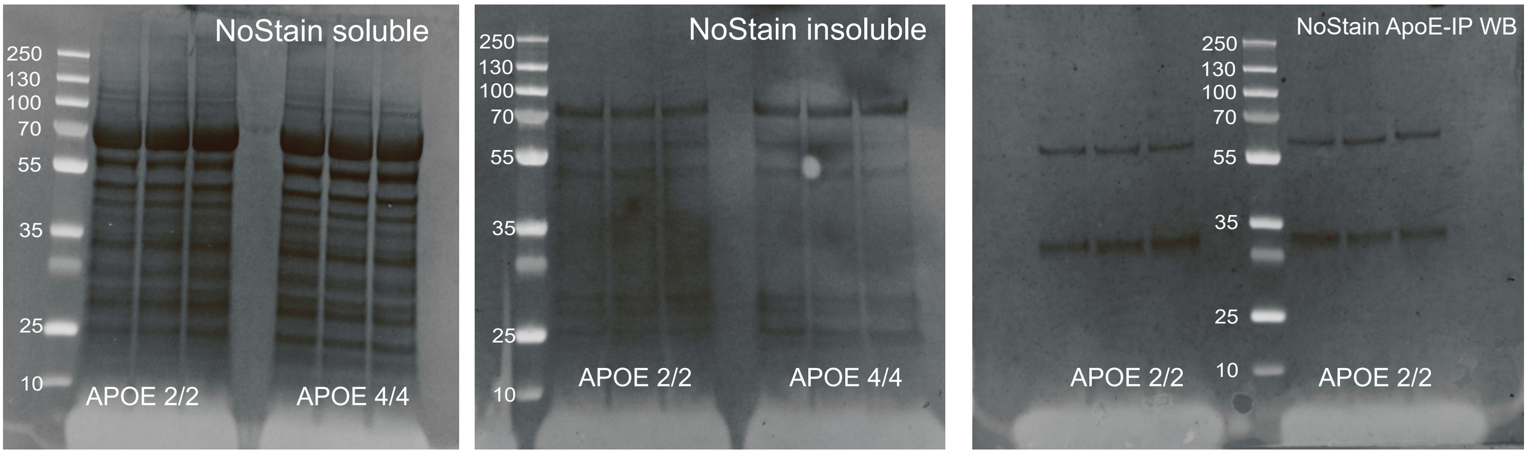

Figure S5. Total protein (NoStain) blots used for normalisation of western blot membranes. Related to Figure 2 f, h and m.

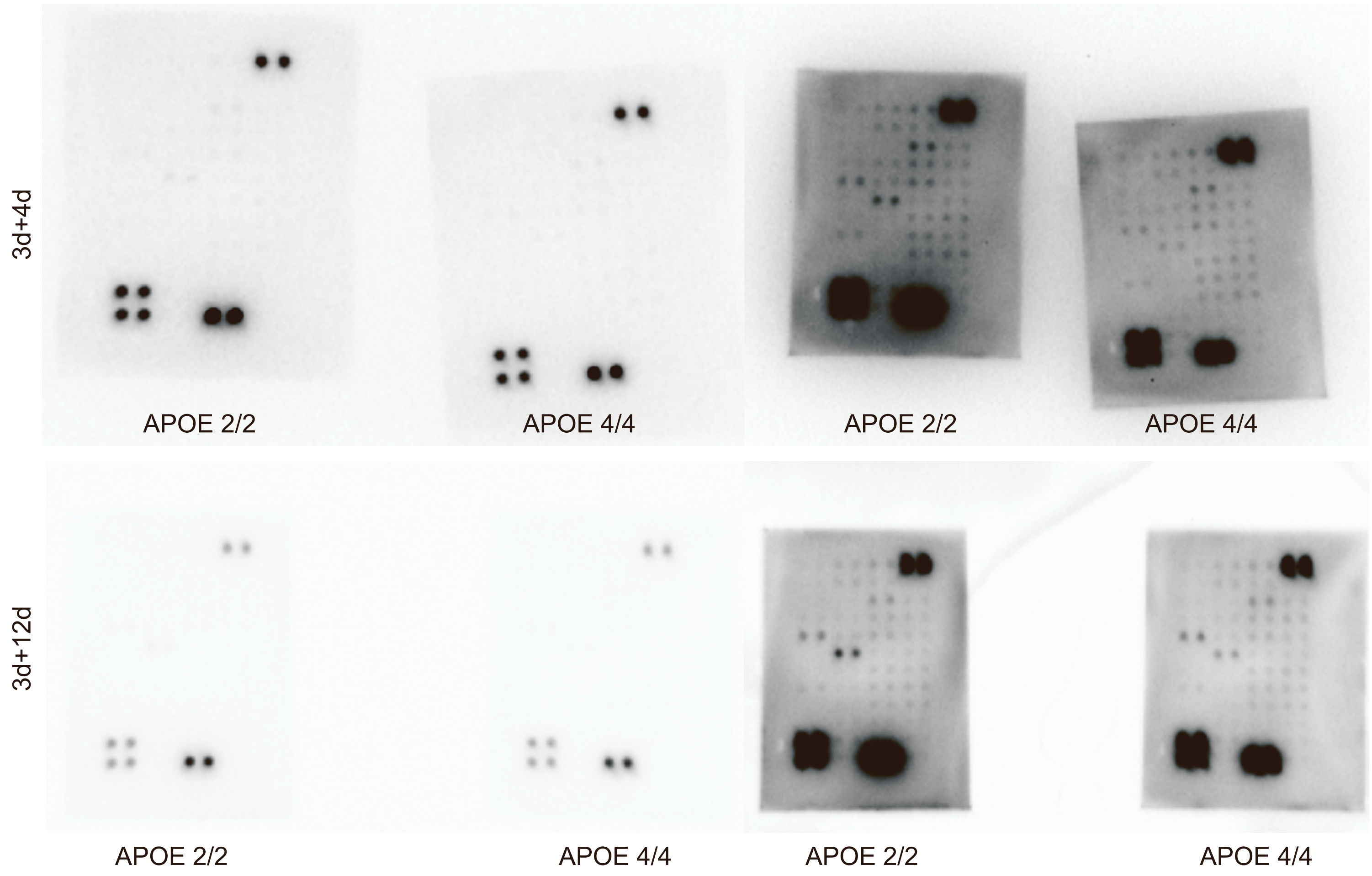

Figure S6. Cytokines in astrocyte medium over time. Related to Figure 3. Cytokine array membranes (original images). Different exposure times were required for detection of all cytokines.

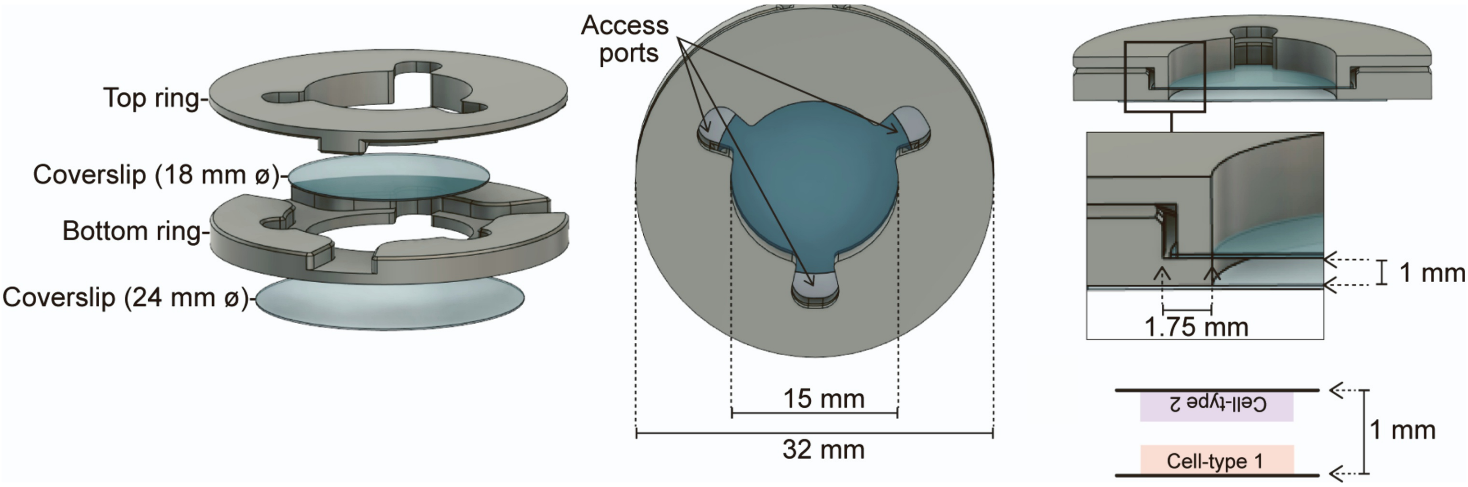

Figure S7. Schematic of the close-chamber culturing system. Related to Figure 5. Two interlocking metal rings (bottom and top) hold a 18 mm coverslip (with *APOE 2/2* or *APOE 4/4* astrocytes) within 1 mm distance of the bottom 24 mm coverslip (with Ctrl9 *APOE 3/3* astrocytes). Access ports allow for medium transport into the space between coverslips.

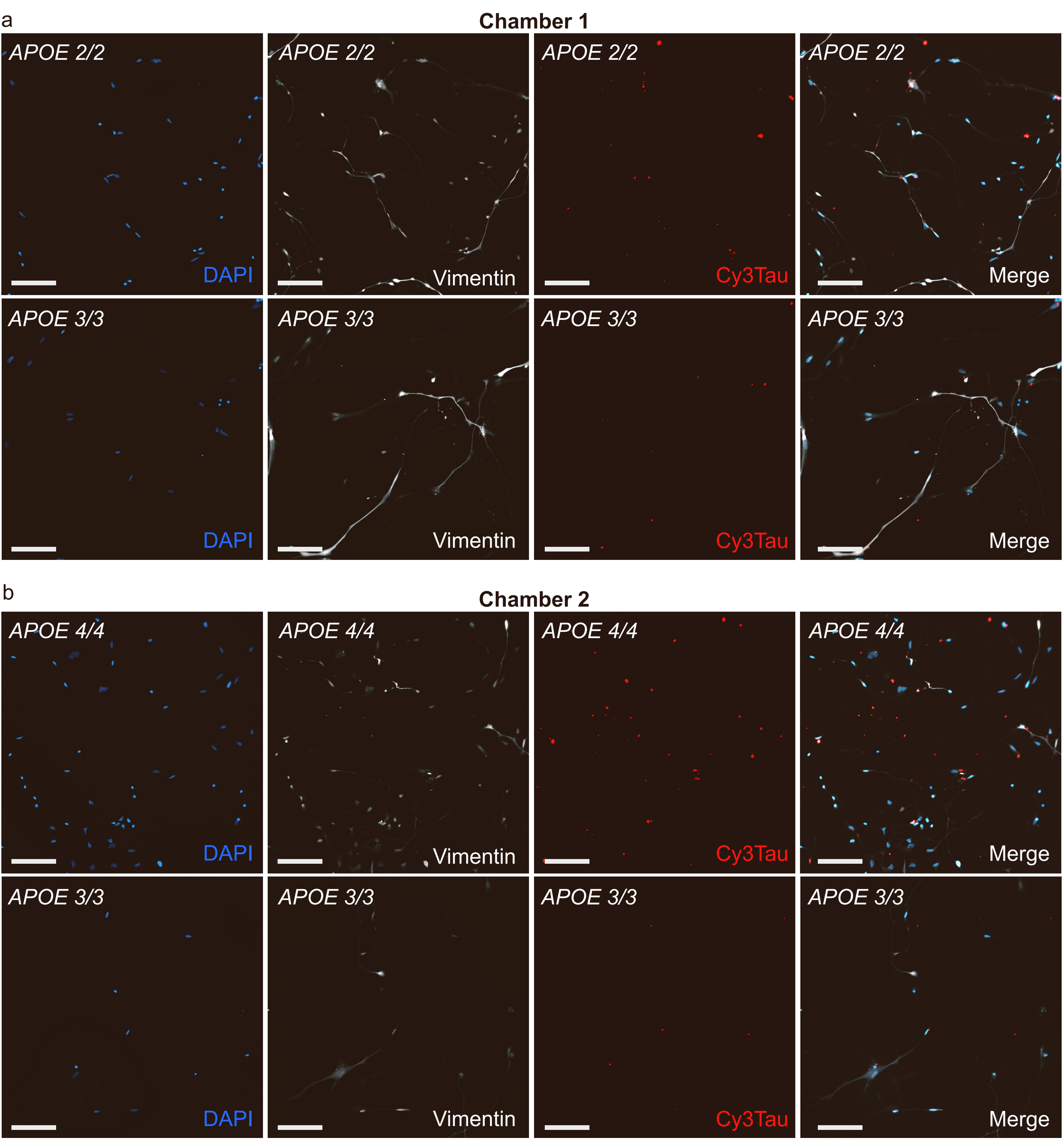

Figure S8. Complete fluorescent images of Cy3Tau in astrocytes. Related to Figure 5b. Cy3Tau exposed (**a**) *APOE 2/2* astrocytes and (**b**) *APOE 4/4* astrocytes after four days in close culture with corresponding *APOE 3/3* astrocytes. All cells were stained for DAPI and Vimentin. Scale bars = 100 μm.

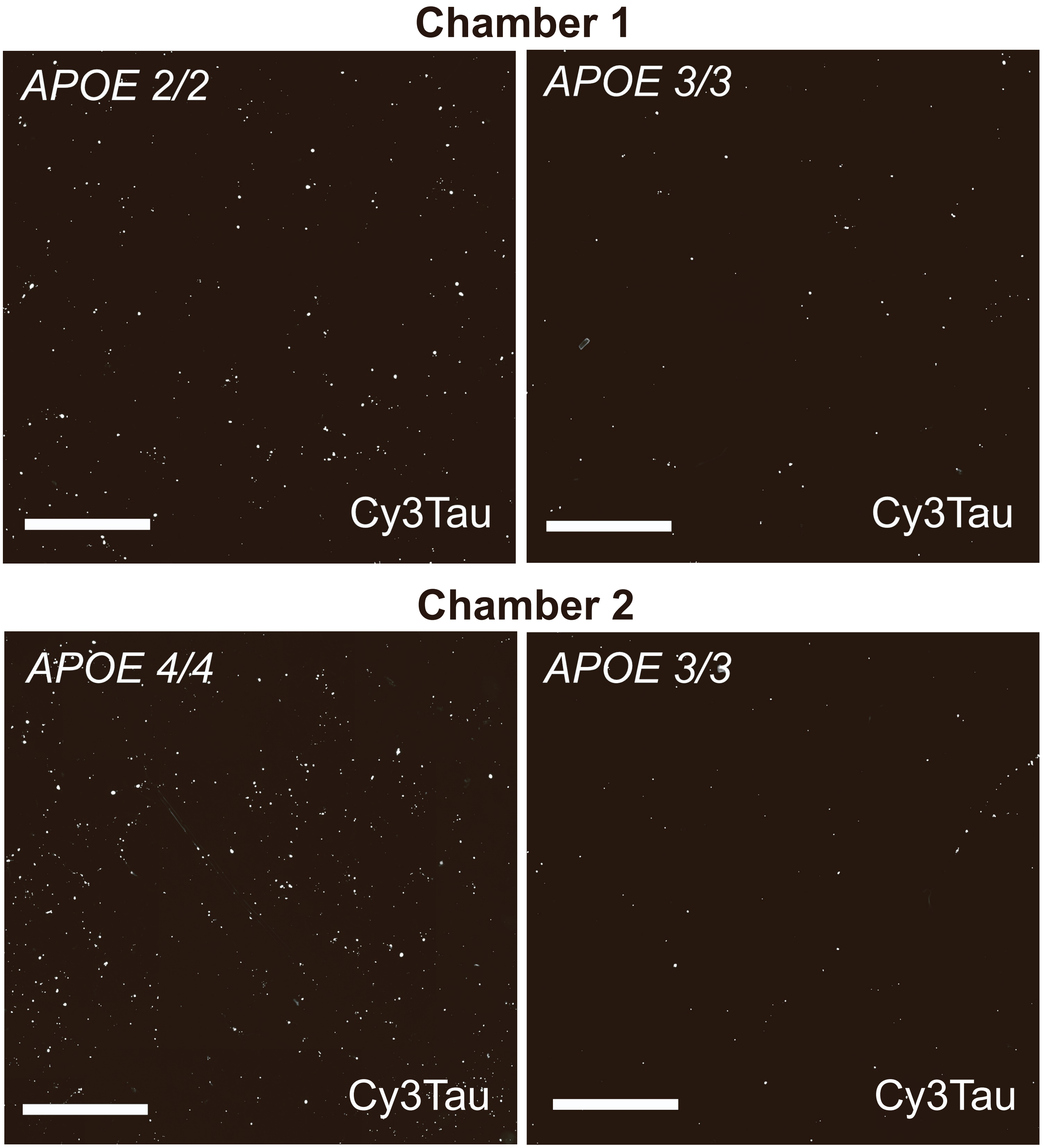

Figure S9. Overview of Cy3Tau (gray-scale) in astrocytes. Related to Figure 5. Chamber 1: *APOE 2/2* and corresponding *APOE 3/3* astrocytes. Chamber 2: *APOE 4/4* and corresponding *APOE 3/3* astrocytes. Each images are taken after four days of tau exposure. Scale bars = 500 μm.

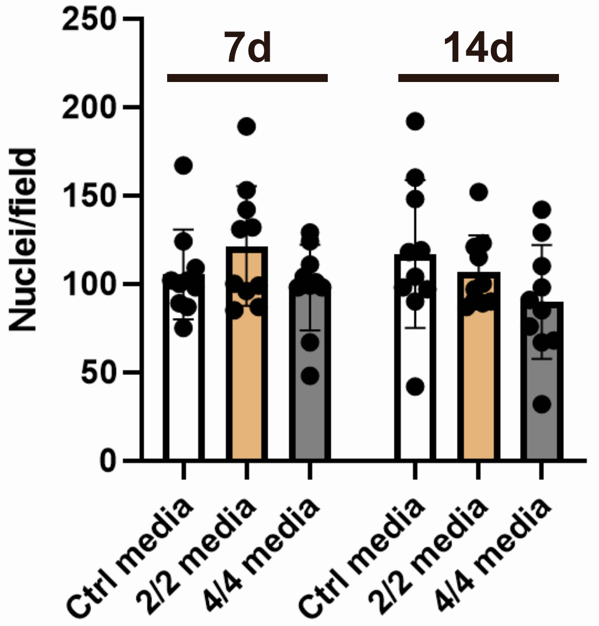

Figure S10. **Cell count (neurons)/field of view. Related to Figure 6.** Average number of neurons/field following exposure to conditioned medium from ctrl, *APOE 2/2* or APOE 4/4 astrocytes.
